# Flipped Quartification: Product Group Unification with Leptoquarks

**DOI:** 10.3390/e26070533

**Published:** 2024-06-21

**Authors:** James B. Dent, Thomas W. Kephart, Heinrich Päs, Thomas J. Weiler

**Affiliations:** 1 Department of Physics, Sam Houston State University, Huntsville, TX 77341, USA; 2 Department of Physics and Astronomy, Vanderbilt University, Nashville, TN 37235, USA; tom.kephart@gmail.com (T.W.K.);; 3 Institut für Physik, Technischen Universität Dortmund, D-44221 Dortmund, Germany; heinrich.paes@uni-dortmund.de

**Keywords:** leptoquarks, beyond the standard model, quantification models, early Universe, thermodynamics, phenomenology

## Abstract

The quartification model is an $SU{\left(3\right)}^{4}$ extension with a bi-fundamental fermion sector of the well-known $SU{\left(3\right)}^{3}$ bi-fundamentalfication model. An alternative “flipped” version of the quartification model is obtained by rearrangement of the particle assignments. The flipped model has two standard (bi-fundamentalfication) families and one flipped quartification family. In contrast to traditional product group unification models, flipped quartification stands out by featuring leptoquarks and thus allows for new mechanisms to explain the generation of neutrino masses and possible hints of lepton-flavor non-universality.

## 1. Introduction

The long-term goal of extending the standard model of particle physics is to develop a model that is more predictive than the standard model and to connect it with physics at higher energy scales. Many people have contributed to the progress toward this goal over the last 50 years, and the effort has continued up until this day (Paul Frampton has been a major contributor to this effort. This article is to acknowledge his work and celebrate his 80th birthday). These scales could be in descending order of energy, the Planck scale at $1.2\times 10^{19}$ GeV, the string scale at about $10^{17}$ GeV, a grand unification scale around $10^{16}$ GeV, or some lower scale where proton decay can be avoided via a partial unification into a product gauge group. The latter two scales are typically set by vacuum expectation values (VEVs) of scalar fields, which give various non-standard model particles their masses but leave the SM fermions and gauge bosons massless. The SM particles themselves remain massless as the energy scale is lowered, until the Higgs scalar electroweak (EW) doubles develop a VEV at 246 GeV. The reason why the EW scale is at such a low energy relative to the Planck scale is one of the key puzzles of the SM, called the hierarchy problem, whose eventual resolution holds great promise for providing a deeper understanding of fundamental physics. Being able to predict other properties of the SM in a systematic way also provides us with the hope that the higher symmetry theory from which the SM descends can eventually be discovered.

Not all the information relevant to extending the SM will necessarily come from particle physics accelerators. The study of particle physics has long been assisted by astronomy, astrophysics, and cosmology. Cosmic rays in particular have played an important role in particle discoveries and searches. The highest energy cosmic rays, while scarce, are still the cause of the highest energy collisions of which we are aware. The origins and acceleration mechanism of these cosmic rays are still unknown, but these rare events are an important window of extreme energies and in turn a potential opportunity to understanding energy scales near unification.

In this paper, we will focus on a particular partial unification into the product gauge group $SU^{4}\left(3\right)$ called a quartification symmetry. Conformal field theories arise naturally as product gauge groups in compactifications of string theories on five dimensional orbifolds (for a review, see Lawrence et al. [1]). Such theories are potentially a way to connect physics at very high energies to physics at energies close to the EW scale. If such a scenario could be fully developed and if it had phenomenological relevance, then it would go a long way to filling out our understanding of a more complete theory of fundamental physics.

Before turning to our particular quartification model, we will first provide some technical remarks to help place it in context. Some phenomenological consequences will be discussed in the final section.

Product group unification schemes include trinification models, refs. [2,3,4,5,6,7,8,9,10,11,12,13,14,15,16,17,18,19,20,21,22,23,24,25,26,27,28,29,30] with gauge group $SU{\left(3\right)}_{L}\times SU{\left(3\right)}_{C}\times SU{\left(3\right)}_{R}$, and quartification models [14,31,32,33,34,35,36,37,38], where the gauge group is extended to $SU{\left(3\right)}_{l}\times SU{\left(3\right)}_{L}\times SU{\left(3\right)}_{C}\times SU{\left(3\right)}_{R}$, and in both classes of models, the fermions are accommodated in bi-fundamental representations. A generic feature of such product group unification schemes is the absence of leptoquarks, i.e., scalar or vector particles that allow transitions between quarks and leptons. In trinification models, leptons are defined by being bi-fundamental under the two SU(3)s that have no color, implying that there are no leptoquarks in such models. Likewise, in traditional quartification models, the SU(3)s are arranged in a way that particles have either $SU{\left(3\right)}_{l}$ or $SU{\left(3\right)}_{C}$ charges, preventing again the occurrence of leptoquarks. This property can be seen as both a blessing and a curse. On the positive side, the absence of transitions between quarks and leptons avoids the occurrence of various processes triggering fast proton decay, yet on the negative side, leptoquarks are attractive components of models for neutrino mass generation [39,40,41] and have been invoked to explain recent anomalies which suggested lepton-flavor non-universality [42,43,44] or both [45,46,47].

Here, we will concentrate on the phenomenology of a new class of quartification models obtained by “flipping” the $SU{\left(3\right)}_{l}$ and $SU{\left(3\right)}_{R}$ groups, which we call “flipped quartification”. In contrast to traditional product-group unification schemes, flipped quartification allows for leptoquarks that are bi-fundamental under the $SU{\left(3\right)}_{C}$ and $SU{\left(3\right)}_{l}$ groups, albeit confined to the third generation, making them less likely of inducing fast proton decays. In addition, the model also singles out the *b* quark as different from all the rest of the SM fermions in that, just above the electro-weak (EW) scale, the EW singlet $b_{R}$ can be in a nontrivial irreducible representation (irrep) of a new gauge group $SU{\left(2\right)}_{l}$, while all the other SM fermions are in $SU{\left(2\right)}_{l}$ singlets. This can happen when the $SU{\left(2\right)}_{l}$ symmetry breaks just above the EW scale where now the $b_{R}$ falls into its usual SM irrep, but with slightly different phenomenology due to nearby $SU{\left(2\right)}_{l}$ effects that the other SM fermions do not feel. This is a fairly conventional but interesting scheme for introducing new physics into the SM.

All quartification models contain an $SU{\left(3\right)}_{l}$ leptonic color sector to realize a manifest quark–lepton symmetry [48,49,50] and must contain at least three families to be phenomenologically viable, plus they contain the new fermions needed to symmetrize the quark and lepton particle content at high energies. Instead of fully quartified models, where all families are quartification families given by

(1) $$3[\left(3\bar{3}11\right)+\left(13\bar{3}1\right)+\left(113\bar{3}\right)+\left(\bar{3}113\right)],$$

we will consider only hybrid models

(2) $$n\left[\left(13\bar{3}1\right)+\left(113\bar{3}\right)+\left(1\bar{3}13\right)\right]+\left(3-n\right)\left[\left(3\bar{3}11\right)+\left(13\bar{3}1\right)+\left(113\bar{3}\right)+\left(\bar{3}113\right)\right].$$

where n>0 families are trinification families and the the remaining 3−n are quartification families. In particular, we concentrate on the n=2 case [36]. One important thing to note here is that both the trinification and quartification family components of these models can be represented by quiver diagrams which are anomaly-free [51]. For models with only bi-fundamental fermions, there are no chiral gauge anomalies since for each 3 there is a $\bar{3}$ with equal and opposite charges. Furthermore, the descendent gauge groups are also guaranteed to be free of anomalies upon breaking the initial gauge symmetry with the ’t Hooft matching conditions [52].

One can derive three family models with appropriate scalar content to permit gauge symmetry breaking to the SM and ultimately to $SU{\left(3\right)}_{C}\times U_{EM}\left(1\right)$ from orbifolded $AdS⊗S^{5}$ (for a review, see [51]). In [53,54], two of us carried out a global search for $\Gamma =Z_{n}$ trinification models with three or more families, and in [36], quartification models of this type were derived from a $\Gamma =Z_{8}$ orbifolded $AdS⊗S^{5}$. We leave the study of the UV completion of the present model for later work.

## 2. Flipped 2 + 1 Quartification Model

Under the original quartification gauge group $SU{\left(3\right)}_{l}\times SU{\left(3\right)}_{L}\times SU{\left(3\right)}_{C}\times SU{\left(3\right)}_{R}$, the representations of the two trinification plus one quartification family model (the 2+1 quartification model of reference [36]) were given by

(3) $$\begin{array}{c} 2\left[\left(13\bar{3}1\right)+\left(113\bar{3}\right)+\left(1\bar{3}13\right)\right]+\left[\left(3\bar{3}11\right)+\left(13\bar{3}1\right)+\left(113\bar{3}\right)+\left(\bar{3}113\right)\right] \end{array}$$

We now “flip” the *R* and *l* designations such that

(4) $$\begin{array}{c} lLCR\to RLCl. \end{array}$$

We are free to cyclically permute the groups and to reverse their order without changing the physics. Thus, we let

(5) $$\begin{array}{c} RLCl\to CLRl \end{array}$$

which allows us to write our new 2+1 flipped quartification model in a form that conforms with the notation of earlier work. Symmetry breaking can easily be arranged with a single adjoint scalar VEV for each of $SU{\left(3\right)}_{L}$ and $SU{\left(3\right)}_{l}$ and a pair of adjoints for $SU{\left(3\right)}_{R}$ such that

(6) $$\begin{array}{ccc} SU{\left(3\right)}_{L} & & \to SU{\left(2\right)}_{L}\times U{\left(1\right)}_{A} \end{array}$$

(7) $$\begin{array}{ccc} SU{\left(3\right)}_{R} & & \to U{\left(1\right)}_{B}\times U{\left(1\right)}_{C} \end{array}$$

(8) $$\begin{array}{ccc} SU{\left(3\right)}_{l} & & \to SU{\left(2\right)}_{l}\times U{\left(1\right)}_{D} \end{array}$$

where the charge operators *A*, *C*, and *D* are of the form diag(1,1,−2) and *B* is of the form diag(1,−1,0). Their weighting in forming weak hypercharge will be provided below.

To be more specific, the symmetry breaking from $SU{\left(3\right)}^{4}$ to $SU{\left(3\right)}_{C}\times SU{\left(2\right)}_{L}\times U{\left(1\right)}^{4}\times SU{\left(2\right)}_{l}$ can be carried out with four adjoints ${\left(1,8,1,1\right)}^{H}$, ${\left(1,1,8,1\right)}^{H}$, ${\left(1,1,8,1\right)}^{H}$ and ${\left(1,1,1,8\right)}^{H}$ which break $SU{\left(3\right)}_{L}$ to $SU{\left(2\right)}_{L}\times U\left(1\right)$, $SU{\left(3\right)}_{R}$ to $U{\left(1\right)}^{2}$ and $SU{\left(3\right)}_{l}$ to $SU{\left(2\right)}_{l}\times U\left(1\right)$, respectively. The remaining U(1)s are broken by appropriately charged singlets of the respective groups. The standard model scalar doublet can come from an ${\left(1,3,1,1\right)}^{H}$ irrep of $SU{\left(3\right)}_{L}$ to yield the standard model Higgs or in the present notation an ${\left(1,2,1,1\right)}^{H}$. No light scalars are required beyond the SM Higgs.

Under the symmetry group $SU{\left(3\right)}_{C}\times SU{\left(2\right)}_{L}\times SU{\left(2\right)}_{l}\times U{\left(1\right)}_{A}\times U{\left(1\right)}_{B}\times U{\left(1\right)}_{C}\times U{\left(1\right)}_{D}$, the first two families decompose as in a standard trinification model,

(9) $$\begin{array}{ccc} & & \left(3\bar{3}11\right)\to {\left(321\right)}_{-1000}+{\left(311\right)}_{2000}\\ & & \left(13\bar{3}1\right)\to {\left(121\right)}_{1-1-10}+{\left(121\right)}_{11-10}+{\left(121\right)}_{1020}+{\left(111\right)}_{-2-1-10}+{\left(111\right)}_{-21-10}+{\left(111\right)}_{-2020}\\ & & \left(\bar{3}131\right)\to {\left(\bar{3}11\right)}_{0110}+{\left(\bar{3}11\right)}_{0-110}+{\left(\bar{3}11\right)}_{00-20} \end{array}$$

while the third family representations become

(10) $$\begin{array}{ccc} & & \left(3\bar{3}11\right)\to {\left(321\right)}_{-1000}+{\left(311\right)}_{2000}\\ & & \left(13\bar{3}1\right)\to {\left(121\right)}_{1-1-10}+{\left(121\right)}_{11-10}+{\left(121\right)}_{1020}+{\left(111\right)}_{-2-1-10}+{\left(111\right)}_{-21-10}+{\left(111\right)}_{-2020}\\ & & \left(113\bar{3}\right)\to {\left(112\right)}_{011-1}+{\left(112\right)}_{0-11-1}+{\left(112\right)}_{00-2-1}+{\left(111\right)}_{0112}+{\left(111\right)}_{0-112}+{\left(111\right)}_{00-22}\\ & & \left(\bar{3}113\right)\to {\left(\bar{3}12\right)}_{0001}+{\left(\bar{3}11\right)}_{000-2}. \end{array}$$

Using the relation

(11) $$\begin{array}{c} Q=T_{3}+Y \end{array}$$

where *Q* is the electric charge, $T_{3}$ is the third component of isospin, and *Y* is the hypercharge, we can determine the hypercharge in terms of the U(1) charges (designated by A,B,C, and *D*) as

(12) $$\begin{array}{c} Y=-\frac{1}{6}A+\frac{1}{2}B-\frac{1}{6}C+\frac{1}{3}D. \end{array}$$

Charged singlets can be used to break $U{\left(1\right)}_{A}\times U{\left(1\right)}_{B}\times U{\left(1\right)}_{C}\times U{\left(1\right)}_{D}$ to the standard weak hypercharge $U{\left(1\right)}_{Y}$, resulting in

(13) $$\begin{array}{ccc} & & \left(3\bar{3}11\right)\to {\left(321\right)}_{\frac{1}{6}}+{\left(311\right)}_{-\frac{1}{3}}\\ & & \left(13\bar{3}1\right)\to {\left(121\right)}_{-\frac{1}{2}}+{\left(121\right)}_{\frac{1}{2}}+{\left(121\right)}_{\frac{1}{2}}+{\left(111\right)}_{0}+{\left(111\right)}_{1}+{\left(111\right)}_{0}\\ & & \left(\bar{3}131\right)\to {\left(\bar{3}11\right)}_{\frac{1}{3}}+{\left(\bar{3}11\right)}_{-\frac{2}{3}}+{\left(\bar{3}11\right)}_{\frac{1}{3}} \end{array}$$

for the first two families, where as usual, each trinification family contains an SM family

(14) $$\begin{array}{ccc} & & Q_{L}^{1(2)}+d{\left(s\right)}_{R}+u{\left(c\right)}_{R}+l_{L}^{1(2)}+e{\left(\mu \right)}_{R}={\left(321\right)}_{\frac{1}{6}}+{\left(\bar{3}11\right)}_{\frac{1}{3}}+{\left(\bar{3}11\right)}_{-\frac{2}{3}}+{\left(121\right)}_{\frac{1}{2}}+{\left(111\right)}_{1} \end{array}$$

plus the following vector-like states:

(15) $$\begin{array}{ccc} & & +{\left(\bar{3}11\right)}_{\frac{1}{3}}+{\left(311\right)}_{-\frac{1}{3}}+{\left(121\right)}_{-\frac{1}{2}}+{\left(121\right)}_{\frac{1}{2}}+{\left(111\right)}_{0}+{\left(111\right)}_{0}. \end{array}$$

The third family in Equation (10) becomes

(16) $$\begin{array}{ccc} & & \left(3\bar{3}11\right)\to {\left(321\right)}_{\frac{1}{6}}+{\left(311\right)}_{-\frac{1}{3}}\\ & & \left(13\bar{3}1\right)\to {\left(121\right)}_{-\frac{1}{2}}+{\left(121\right)}_{\frac{1}{2}}+{\left(121\right)}_{\frac{1}{2}}+{\left(111\right)}_{0}+{\left(111\right)}_{1}+{\left(111\right)}_{0}\\ & & \left(113\bar{3}\right)\to {\left(112\right)}_{0}+{\left(112\right)}_{-1}+{\left(112\right)}_{0}+{\left(111\right)}_{1}+{\left(111\right)}_{0}+{\left(111\right)}_{1}\\ & & \left(\bar{3}113\right)\to {\left(\bar{3}12\right)}_{\frac{1}{3}}+{\left(\bar{3}11\right)}_{-\frac{2}{3}} \end{array}$$

which we rearrange in a more suggestive form

(17) $$\begin{array}{ccc} & & {\left(321\right)}_{\frac{1}{6}}+{\left(\bar{3}11\right)}_{-\frac{2}{3}}+{\left(121\right)}_{\frac{1}{2}}+{\left(111\right)}_{1}\\ & & +{\left(\bar{3}12\right)}_{\frac{1}{3}}+\left[{\left(112\right)}_{0}+{\left(112\right)}_{0}\right]+\left[{\left(112\right)}_{-1}+{\left(111\right)}_{1}+{\left(111\right)}_{1}\right]+{\left(111\right)}_{0}\\ & & +{\left(311\right)}_{-\frac{1}{3}}+\left[{\left(121\right)}_{-\frac{1}{2}}+{\left(121\right)}_{\frac{1}{2}}\right]+{\left(111\right)}_{0}+{\left(111\right)}_{0}. \end{array}$$

The first line of Equation (17) contains an SM family except that $b_{R}$ is missing. The second line contains states in nontrivial $SU{\left(2\right)}_{l}$ irreps and their natural partners, and the last line contains the remaining states.

In order to complete the third SM family, a ${\left(\bar{3}11\right)}_{\frac{1}{3}}$ from the second line must be moved to the first line. To perform this, we can either (i) break $SU{\left(2\right)}_{l}\to 0$ at a scale $M_{ssb}$ or (ii) arrange to have the gauge coupling of $SU{\left(2\right)}_{l}$ run to large values, where at some scale $\Lambda_{l}$ this group becomes confining. We expect the lower bounds on $M_{ssb}$ and $\Lambda_{l}$ to be similar.

To complete the third family via spontaneous symmetry breaking, we introduce a scalar $SU{\left(2\right)}_{l}$ doublet ${\left(1,1,2\right)}_{0}$ whose VEV breaks $SU{\left(2\right)}_{l}$ completely so that ${\left(\bar{3}12\right)}_{\frac{1}{3}}\to {\left(\bar{3}11\right)}_{\frac{1}{3}}+{\left(\bar{3}11\right)}_{\frac{1}{3}}$. One of these two irreps can be identified with the $b_{R}$, hence completing the third family in the first line of Equation (17). The other we identify as the $b_{R}^{\prime }$, which pairs with the ${\left(311\right)}_{-\frac{1}{3}}$ in the third line of Equation (17). The chargeless $SU{\left(2\right)}_{l}$ doublet leptonic states in the second line of Equation (17) also split into singlets, while the charge −1 doublet $SU{\left(2\right)}_{l}$ irreps split so that they can pair with the charge +1 singlet leptons in that line. Writing Equation (17) after the symmetry breaking, where we have moved half the split ${\left(\bar{3}12\right)}_{\frac{1}{3}}$ irrep into the first line and the other half into the third line gives

(18) $$\begin{array}{ccc} & & {\left(321\right)}_{\frac{1}{6}}+{\left(\bar{3}11\right)}_{\frac{1}{3}}+{\left(\bar{3}11\right)}_{-\frac{2}{3}}+{\left(121\right)}_{\frac{1}{2}}+{\left(111\right)}_{1}\\ & & +\left[{\left(111\right)}_{0}+{\left(111\right)}_{0}+{\left(111\right)}_{0}+{\left(111\right)}_{0}\right]+\left[{\left(111\right)}_{-1}+{\left(111\right)}_{-1}+{\left(111\right)}_{1}+{\left(111\right)}_{1}\right]+{\left(111\right)}_{0}\\ & & +{\left(\bar{3}11\right)}_{\frac{1}{3}}+{\left(311\right)}_{-\frac{1}{3}}+\left[{\left(121\right)}_{-\frac{1}{2}}+{\left(121\right)}_{\frac{1}{2}}\right]+{\left(111\right)}_{0}+{\left(111\right)}_{0} \end{array}$$

The SSB has yielded a standard third family in the first line, states with identical charges to the extra trinification family in the third line, plus the new extra states of a quartification family in the second line. In the following, we concentrate on the properties of the *b* quark.

Note that all three families have an extra $d^{\prime }$ type quark in ${\left(311\right)}_{-\frac{1}{3}}+{\left(\bar{3}11\right)}_{\frac{1}{3}}$, which is typical of all trinification or $E_{6}$ models. For the first two families, they are in vector-like representations, so these particles can acquire mass at a high scale, and we will not discuss them further. However, in the third family, the $b^{\prime }$ can not acquire a mass until $SU{\left(2\right)}_{l}$ is broken. Thus, the third family $b^{\prime }$ is phenomenologically more interesting.

As we are completing the third family via spontaneous symmetry breaking, at some scale *M*, then the only chiral fermions below that scale are in the standard families. All the rest are vector-like; see Equation (18), and obtain masses around the scale $M_{ssb}$.

## 3. Phenomenological Implications

For spontaneous symmetry breaking of $SU{\left(2\right)}_{l}$, we find a phenomenology that is a straightforward extension of the SM: it contains the normal SM particle content in the first two families plus their trinification extension. The third quartified family contains a third normal family, its extended trinification content, plus the remaining extended quartification content composed of two $SU{\left(2\right)}_{L}$ singlet unit electric charged leptons and five Weyl neutrinos, some of which can be paired up after SSB.

*Extended $Z^{\prime }$ bosons sector:* The gauge group of our $SU{\left(3\right)}^{4}$ flipped quartification model is rank 8, while the standard model is rank 4, so FQ has four additional uncharged $Z^{\prime }$-like gauge bosons. Depending on how the spontaneous symmetry breaking proceeds, their masses can range from the initial $SU{\left(3\right)}^{4}$ breaking scale down to the current experimental limit on $Z^{\prime }$ masses. The four $Z^{\prime }$ masses can all be different within these bounds. We have yet to explore the full parameter space of allowed FQ models, so we are reluctant to give the full set of constraints on the $Z^{\prime }$s yet, but we hope to come back to this interesting phenomenological question in future work.

*Leptoquarks, Hints for Lepton-Flavor-Non-Universality, and the Muon Anomaly:* A characteristic property of the “flipping” in the order of the quartification gauge group within the present construction is the likelihood of the presence of light leptoquarks. This could be realized by the scalar or vector representation that couple terms in the bi-fundamental fermions that are nontrivial in $SU{\left(3\right)}_{C}$ with those in $SU{\left(2\right)}_{L}$. (Bileptons and/or biquarks could also be present. For a full classification, see [55].) Leptoquarks have been a popular possibility to explain the recent *b*-physics anomalies pointing at lepton-flavor non-universality (see, e.g., [42,56]), though recent results from the LHCb collaboration are consistent with Standard Model predictions [57,58]. Regardless of these recent collider results, leptoquarks have a rich phenomenology that will continue to be explored in BSM scenarios of flavor physics and neutrino mass origins, to name a few (see, for example, ref. [59] for a review of the varieties of leptoquark phenomenology). For the most recent experimental results on leptoquarks, see the publications from ATLAS [60,61,62] and CMS [63,64,65].

There is another interesting leptoquark possibility in the flipped quartification model discussed above. Since the third family has $SU{\left(3\right)}_{l}$ quantum numbers, there is also the possibility of vector leptoquark contributions from this sector. Likewise, there are potential scalar $SU{\left(3\right)}_{l}$ leptoquarks if we were to add the appropriate scalar irreps.

An interesting result is Fermilab’s recent confirmation of an anomalous result for the magnetic moment of the muon [66]. In [67], it had been shown that the anomalous magnetic moment of the muon could be explained by adding a vector-like doublet plus a scalar singlet to the particle content of the SM. In the present model the states ${\left(112\right)}_{0}+{\left(112\right)}_{0}$ in the second line of Equation (17) can play the role of the vector-like doublet. See also [68].

Finally, while the model we have presented can be used to focus on *B* physics, other models in this class can be used to single out one or more right-handed charge $-\frac{1}{3}$ quarks. Then right-handed quarks are made to fall into flipped quartification families, while the remaining right-handed charge $-\frac{1}{3}$ quarks remain in trinification families. Future work can potentially lead to a whole class of models similar to flipped quartification where one or more fermions are singled out to differ from other normal family members, hence providing a rich and interesting BSM phenomenology.

Changing the model of particle physics to the FQ model has implications for astrophysics and cosmology. For instance, let us compare the thermodynamics of the early Universe for SU(5) unification with that of the FQ model. When the SU(5) gauge group breaks at a high scale, there is typically a first-order phase transition that produces magnetic monopoles and also causes inflation, after which the Universe evolves adiabatically until SM symmetry breaking. By contrast, the FQ model can undergo many phase transitions and have a much more complicated thermodynamics. Starting from $SU{\left(3\right)}^{4}$ and identifying one SU(3) group as color, the other three can each break to SU(2)×U(1), and then two of the SU(2)s can break to U(1)s. At this stage, the gauge group is $SU\left(3\right)\times SU\left(2\right)\times U{\left(1\right)}^{5}$. Then the $U{\left(1\right)}^{5}$ part must break to $U{\left(1\right)}_{Y}$. These symmetry breakings can occur sequentially or some can happen concurrently. Some of these phase transitions can be first order, leading to particle production and entropy production. All the symmetry breakings that produce a U(1) produce monopoles. All U(1) breakings can produce cosmic strings. Monopole–antimonopole pairs can annihilate if they are at the end of a string. It is clear that the thermodynamics of the early Universe for FQ models can have a wide variety of implications for astro-particle physics and cosmology, all worthy of future study.

## 4. Summary

In this paper, we have discussed a novel class of quartification models with the curious feature that they—in contrast to traditional product group unification schemes—allow for the occurrence of leptoquarks and thus an interesting phenomenology for neutrino mass generation and other beyond-the-Standard-Model processes, such as lepton-flavor non-universality.

There are many FQ model building options to consider. The FQ model should be thought of as string-inspired, not string-derived. This allows us more leeway to explore potential phenomenologies; e.g., there are multiple ways to break the extra U(1)s to the required hypercharge $U{\left(1\right)}_{Y}$. Since it is a linear combination of U(1)s coming from different SU(3)s, this requires charged scalar representations living in multiple SU(3)s, e.g., bi-fundamental Higgses like ${\left(1,1,3,\bar{3}\right)}_{H}$, etc. Such representations would arise naturally in, say, an N=1 SUSY $S^{5}/Z_{4}$ orbifolding.

From a purely phenomenological perspective, we could use any Higgs we like. Different choices lead to different symmetry breaking scenarios with different mass scales for the breaking. The breaking of $SU{\left(3\right)}^{4}$ to the non-abelian part of the standard model gauge group is easily accomplished with scalar octets, so that part of the phenomenology should be straightforward.

The $SU{\left(3\right)}^{4}$ scale is set by how much the gauge couplings need to run to get to their SM values. The initial values of the $SU{\left(3\right)}^{4}$ gauge couplings can be set by the fact that these SU(3)s can be in diagonal subgroups of some larger group, $SU{\left(3\right)}^{p}\times SU{\left(3\right)}^{q}\times SU{\left(3\right)}^{r}\times SU{\left(3\right)}^{s}$, where the ratios of p/q/r/s set the initial values of these FQ SU(3) couplings [69]. Consequently we can raise or lower the initial values by changing the string model orbifolding group, $\Gamma =Z_{n}$, where n=p+q+r+s.

This multitude of possibilities makes the FQ models a rich source of phenomenology, but it will take a dedicated effort to explore all the parameter space and to optimize the model with respect to new phenomenology and simple economical patterns of SSB. For this reason we have added an overview of phenomenological possibilities and SSBs in the final section of the manuscript but have not committed to a specific model. We are reluctant to make such a choice here before we feel comfortable with having accomplished all we can to select the best model. Hence, we believe this is better left to future work once we have fully explored all the options.

Within this model, we found that the third family of the Standard Model can be completed via spontaneous symmetry breaking of an unbroken $SU{\left(2\right)}_{l}$. Completion via spontaneous symmetry breaking leads to interesting leptoquarks and bileptons coupled only to the third family, which can potentially avoid proton decay but still extend standard model phenomenology. We leave the details to future work. Beyond the possibility of having leptoquarks, if the $SU{\left(2\right)}_{l}$ group becomes confining at a high scale, it leads to a possible composite *b* quark. However, we have yet to build a successful phenomenology from this prospective and leave further considerations along these lines to future work.

## Data Availability

The original contributions presented in the study are included in the article, further inquiries can be directed to the corresponding author.
